# Evaluating of the association between ABO blood groups and coronavirus disease 2019 (COVID-19) in Iraqi patients

**DOI:** 10.1186/s43042-020-00097-x

**Published:** 2020-09-11

**Authors:** Ali H. Ad’hiah, Risala H. Allami, Raghdan H. Mohsin, Maha H. Abdullah, Ali J. R. AL-Sa’ady, Mustafa Y. Alsudani

**Affiliations:** 1 Tropical-Biological Research Unit, College of Science, University of Baghdad, Baghdad, Iraq; 2 College of Biotechnology, Al-Nahrain University, Baghdad, Iraq; 3 College of Agriculture, University of Basrah, Basrah, Iraq; 4 Biotechnology Department, College of Science, University of Baghdad, Baghdad, Iraq; 5 Basrah Health Office, Ministry of Health and Environment, Baghdad, Iraq

**Keywords:** COVID-19, ABO blood group, Age, Gender

## Abstract

**Background:**

Susceptibility to the pandemic coronavirus disease 2019 (COVID-19) has recently been associated with ABO blood groups in patients of different ethnicities. This study sought to understand the genetic association of this polymorphic system with risk of disease in Iraqi patients. Two outcomes of COVID-19, recovery and death, were also explored. ABO blood groups were determined in 300 hospitalized COVID-19 Iraqi patients (159 under therapy, 104 recovered, and 37 deceased) and 595 healthy blood donors. The detection kit for 2019 novel coronavirus (2019-nCoV) RNA (PCR-Fluorescence Probing) was used in the diagnosis of disease.

**Results:**

Mean age was significantly increased in patients compared to controls (49.8 ± 11.7 vs. 28.9 ± 6.6 years; *p* < 0.001). A similar observation was made in recovered (42.1 ± 10.4 vs. 28.9 ± 6.6 years; *p* < 0.001) and deceased (53.6 ± 9.7 vs. 28.9 ± 6.6 years; *p* < 0.001) cases. The mean age was also significantly increased in deceased cases compared to recovered cases (53.6 ± 9.7 vs. 42.1 ± 10.4 years; *p* < 0.001). There were gender-dependent differences in COVID-19 prevalence. The percentage of COVID-19 was higher in males than in females (all cases: 59.7 vs. 40.3%; recovered cases: 55.8 vs. 44.2%). Such male-gender preponderance was more pronounced in deceased cases (67.6 vs. 32.4%). Logistic regression analysis revealed that groups AB and B + AB were significantly associated with increased risk to develop COVID-19 (OR = 3.10; 95% CI 1.59–6.05; *pc* = 0.007 and OR = 2.16; 95% CI 1.28–3.63; *pc* = 0.028, respectively). No ABO-associated risk was observed in recovered cases. On the contrary, groups A (OR = 14.60; 95% CI 2.85–74.88; *pc* = 0.007), AB (OR = 12.92; 95% CI 2.11–79.29; *pc* = 0.042), A + AB (OR = 14.67; 95% CI 2.98–72.33; *pc* = 0.007), and A + B + AB (OR = 9.67; 95% CI 2.02–46.24; *pc* = 0.035) were associated with increased risk of death in deceased cases.

**Conclusions:**

The findings of this study suggest that group AB may be a susceptibility biomarker for COVID-19, while group A may be associated with increased risk of death.

## Background

Severe acute respiratory syndrome coronavirus 2 (SARS-CoV-2) is the strain of coronavirus that causes coronavirus disease 2019 (COVID-19) [1]. Since the emergence of the first case of COVID-19 in December 2019 (Wuhan, China), the infection has become pandemic spreading to more than 300 nations. Up to the fifth of June 2020, 11,241,655 total cases have been reported with a mortality rate of 4.7% (total deaths = 530,668 cases). The corresponding figures for Iraq were 58,354 and 4.1% (total deaths = 2368), respectively [2]. Up-to-date, there has been no effective therapy or vaccine. Further, there has been no specific biomarker for the disease. Risk factors that have been determined were mostly related to general clinical observations; for instance, age, gender, and chronic diseases [3]. However, ABO blood groups have been recently introduced as a genetic system that may influence susceptibility to COVID-19 [4, 5].

The ABO blood group antigens are cell-surface glycoproteins present principally on erythrocytes and on a variety of other cell lines and tissues. They are indirectly encoded by a single locus on chromosome 9 (9q34.1-q34.2) [6]. At the ABO locus, two codominant functional alleles encode blood group A and B transferases (*A* and *B* alleles, respectively). A third recessive *O* allele encodes enzymatically inactive proteins that have no activity of either of the transferases. Accordingly, four basic ABO phenotypes are recognized; A, B, AB, and O [7]. ABO alleles and phenotypes are frequent targets for epidemiological and anthropological studies because they are genetically determined traits, and their polymorphic expression is documented among populations so far investigated. Frequency of these alleles and phenotypes is race-dependent and the major racial populations (Caucasians, Orientals, and Negros) show great variations [8]. Further, different disease-association studies have disclosed the significance of ABO blood groups as genetic risk factors for different human diseases (viral, bacterial, fungal, parasitic, and malignant diseases) [9].

With respect to COVID-19, recent studies have associated ABO blood groups with susceptibility to disease. In these studies, group A, B, or AB was suggested to act as a risk factor for the infection, while group O was associated with a decreased risk in most populations investigated [4, 5, 10]. Before that, an epidemiological study was conducted during the SARS-CoV outbreak (2002–2003) on hospital workers who contracted the infection. The analysis revealed that workers with group O were less likely to become infected with SARS-CoV compared to non-O workers [11].

In line with these finding, the present preliminary study sought to understand the genetic association of ABO blood groups with susceptibility to COVID-19 in Iraqi patients. Two outcomes of the disease, recovery and death, were also explored in this context. To the best of our knowledge, this is the first study in Iraqi patients.

## Methods

### Populations studied

**Fig. 1:**
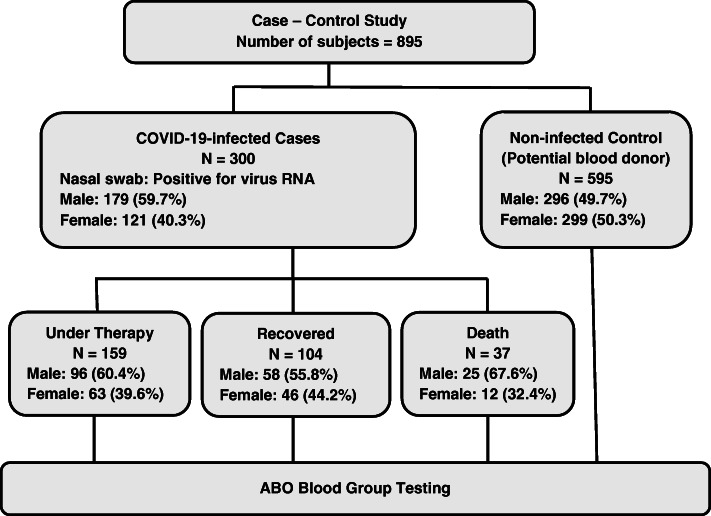
Flowchart showing experimental design of study

**Fig. 2:**
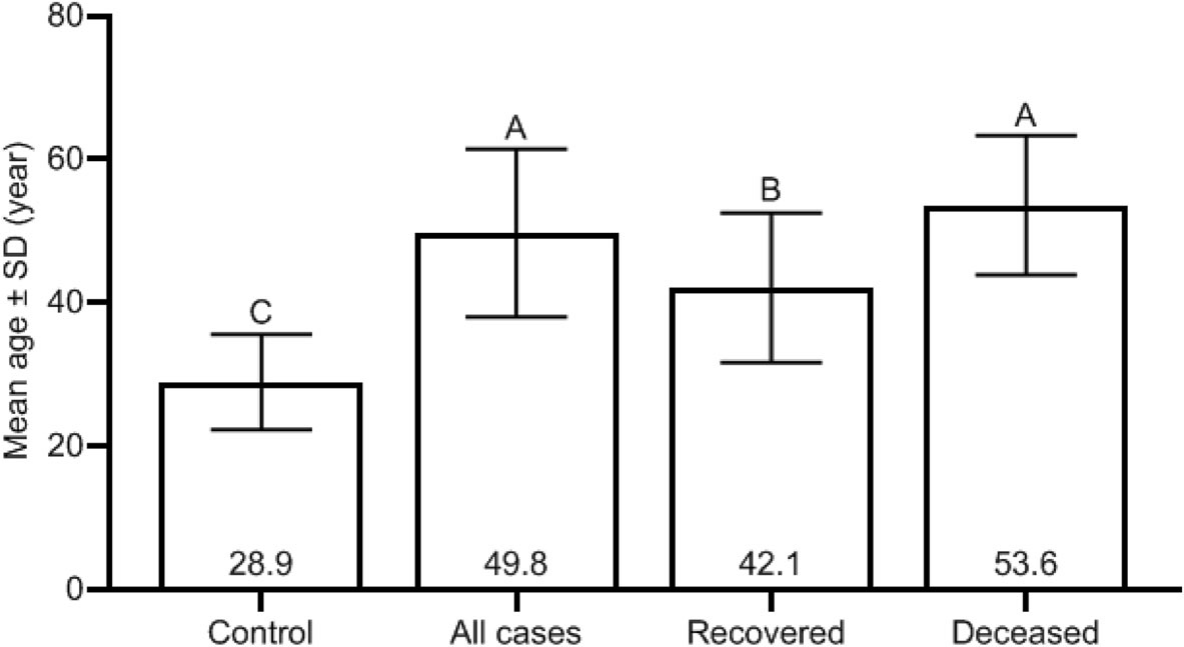
Coronavirus disease 2019 cases and control distributed according to mean age. Different uppercase letters represent significant difference between means of bars (*p* value < 0.001), while similar letters represent no significant difference between means of bars (*p* value > 0.05) (Duncan multiple range test)

During June 2020, a case-control study was conducted after obtaining the participants written informed consent and approval of the Ethics Committee at the Iraqi Ministry of Health and Environment (N268 on 31 May 2020). Three hundred cases were recruited from hospitals in Baghdad; 159 under therapy, 104 recovered, and 37 deceased cases. Upon admission to hospital (24–72 h), nasal swabs of patients were examined using the detection kit for 2019 novel coronavirus (2019-nCoV) RNA (PCR-Fluorescence Probing) following instructions of manufacturer (Da An Gene Co., Ltd. of Sun Yat-sen University, China). Enrolled patients were those admitted to the hospitals with symptoms suggestive of COVID-19 and had laboratory-confirmed infection as determined by the aforementioned kit (inclusion criteria). Patients with suspected COVID-19 and were negative for the test were excluded from the study. A control sample of 595 individual (potential blood donors) were also included in the study, and their serum profile for anti-virus antibodies was negative (Central Blood Bank, Baghdad). Age and gender distributions of patients and controls are given in Figs. 1 and 2 and Table 1.

**Table 1:** Mean age of coronavirus disease 2019 cases and controls distributed according to gender

Gender	Mean age ± SD (year)			
	Controls	All cases	Recovered	Deceased
Male	29.3 ± 6.9^C^	49.7 ± 12.3^A^	41.7 ± 11.1^B^	54.0 ± 8.8^A^
Female	28.6 ± 6.3^C^	49.9 ± 10.9^A^	42.6 ± 9.7^B^	52.7 ± 11.7^A^
*p* value	0.197	0.885	0.665	0.708

*SD* standard deviation, *p* least significant difference probability for comparison between males and females

Different superscript uppercase letters represent significant difference between means in rows (*p* value < 0.001), while similar letters represent no significant difference between means in rows (*p* value > 0.05) (Duncan multiple range test)

### Statistical analysis

ABO blood group alleles and phenotypes were given as numbers and percentage frequencies. Estimation of allele frequencies and Hardy-Weinberg equilibrium (HWE) testing were carried out using the software S2 ABOestimator (http://webpages.fc.ul.pt/~pjns/Soft/ABOestimator). Logistic regression analysis (adjusted for age and gender) was employed to assess the genetic association of ABO blood group with COVID-19 infection. It was expressed as odds ratio (OR) and 95% confidence interval (CI). The least significant difference (LSD) or Duncan multiple range test assessed the significant differences between ages, which were given as mean ± standard deviation (SD). A probability (*p*)-value ≤ 0.05 was considered statistically significant after applying Bonferroni correction (*pc*). The statistical package IBM SPSS Statistics 25.0 (Armonk, NY: IBM Corp.) was used to carry out these analyses.

## Results

### Age and gender

Mean age was significantly increased in COVID-19 cases compared to controls (49.8 ± 11.7 vs. 28.9 ± 6.6 years; *p* < 0.001). A similar observation was made in recovered (42.1 ± 10.4 vs. 28.9 ± 6.6 years; *p* < 0.001) and deceased (53.6 ± 9.7 vs. 28.9 ± 6.6 years; *p* < 0.001) cases. The mean age was also significantly increased in deceased cases compared to recovered cases (53.6 ± 9.7 vs. 42.1 ± 10.4 years; *p* < 0.001) (Fig. 2). There were gender-dependent differences in the disease prevalence. The percentage of COVID-19 was higher in males than in females (all cases 59.7 vs. 40.3%; recovered cases 55.8 vs. 44.2%). Such male-gender preponderance was more pronounced in deceased cases (67.6 vs. 32.4%). However, the mean age showed no significant variation between males and females in each group of patients or controls (Table 1).

### ABO phenotypes and alleles

Frequencies of A, B, AB, and O blood group phenotypes showed significant variations between COVID-19 patients (all cases, recovered, and deceased) and controls (*pc* value = 8.8 × 10^−4^, 0.012 and 1.5 × 10^−6^, respectively) (Table 2). Estimating ABO allele frequencies revealed that *r*[O] was the most frequent allele in controls (allele frequency = 0.577). It was also most frequently encountered in all and recovered cases, but with a lower frequency compared to controls (0.465 and 0.526 vs. 0.577, respectively). Such profile was different in deceased cases, and *p*[A] was the most frequent allele (allele frequency = 0.601) (Table 3).

**Table 2:** Phenotype frequencies of ABO blood groups among coronavirus disease 2019 cases and controls

Group	ABO blood group; *N* (%)				*p* value	*pc* value
	A	B	AB	O		
Controls (*N* = 595)	186 (31.3)	142 (23.9)	62 (10.4)	205 (34.4)		
All cases (*N* = 300)	86 (28.7)	80 (26.7)	59 (19.6)	75 (25.0)	2.3 × 10^−4^	8.8 × 10^−4^
Recovered cases (*N* = 104)	19 (18.3)	27 (26.0)	22 (21.1)	36 (34.6)	0.003	0.012
Deceased cases (*N* = 37)	20 (54.1)	3 (8.1)	12 (32.4)	2 (5.4)	3.7 × 10^−7^	1.5 × 10^−6^

*p* Pearson chi-squared test probability compared to controls, *pc* Bonferroni corrected *p*

**Table 3:** Gene frequency of ABO blood group alleles among coronavirus disease 2019 cases and controls

Group	Gene frequency (standard deviation)			Hardy-Weinberg equilibrium		
	*p*[A]	*q*[B]	*r*[O]	Log likelihood	Chi-square	*p* value
Controls	0.235 (0.013)	0.188 (0.012)	0.577 (0.016)	− 779.7	2.717	0.993
All cases	0.274 (0.020)	0.261 (0.019)	0.465 (0.023)	− 418.4	11.197	0.001
Recovered cases	0.212 (0.030)	0.261 (0.033)	0.526 (0.038)	− 148.4	16.245	< 0.001
Deceased cases	0.601 (0.072)	0.218 (0.051)	0.181 (0.068)	− 40.1	1.756	0.185

*p* probability

### Logistic regression analysis

Logistic regression analysis revealed that groups AB and B + AB versus groups O (reference category) were associated with a significantly increased risk to develop COVID-19 (OR = 3.10; 95% CI 1.59–6.05; *pc* value = 0.007 and OR = 2.16; 95% CI 1.28–3.63; *pc* value = 0.028, respectively). No ABO-associated risk was observed in recovered cases. However, a significantly increased risk of death in COVID-19 cases was associated with groups A, AB, A + AB, and A + B + AB (*pc* value = 0.007, 0.042, 0.007, and 0.035, respectively). The ORs these associations were 14.60 (95% CI 2.85–74.88), 12.92 (95% CI 2.11–79.29), 14.67 (95% CI 2.98–72.33), and 9.67 (95% CI 2.02–46.24), respectively (Table 4).

**Table 4:** Genetic association of ABO blood group phenotypes with coronavirus disease 2019

Comparison	Phenotype	OR	95% CI	*p* value	*pc* value
All cases vs. controls	O	Reference			
	A	1.46	0.84–2.56	0.178	1.000
	B	1.75	0.98–3.13	0.059	0.413
	AB	3.10	1.59–6.05	0.001	0.007
	A + AB	1.87	1.12–3.12	0.016	0.112
	B + AB	2.16	1.28–3.63	0.004	0.028
	A + B + AB	1.83	1.14–2.94	0.013	0.091
Recovered cases vs. controls	O	Reference			
	A	0.76	0.38–1.49	0.417	1.000
	B	1.05	0.55–2.02	0.877	1.000
	AB	1.91	0.91–4.00	0.087	0.609
	A+AB	1.11	0.62–1.96	0.731	1.000
	B+AB	1.32	0.75–2.3	0.342	1.000
	A+B+AB	1.09	0.65–1.84	0.750	1.000
Deceased cases vs. controls	O	Reference			
	A	14.60	2.85–74.88	0.001	0.007
	B	2.07	0.29–14.78	0.469	0.609
	AB	12.92	2.11–79.29	0.006	0.042
	A + AB	14.67	2.98–72.33	0.001	0.007
	B + AB	6.30	1.21–32.81	0.029	0.203
	A + B + AB	9.67	2.02–46.24	0.005	0.035

*OR* odds ratio, *CI* confidence interval, *p* logistic regression analysis probability adjusted for age and gender, *pc* Bonferroni corrected *p*

## Discussion

Among the 300 COVID-19, males were more frequently encountered than females, especially in deceased cases. Previous studies also demonstrated that male cases outnumbered female cases, and males tended to have a more severe disease or at a critical status of illness. Further, males have been demonstrated to have 2.4 times risk of death compared to females [12–15]. There is no documented explanation for such gender preponderance of COVID-19 among males. However, it has been recently explored that such gender difference can be attributed to some comorbidities that indirectly increase the risk of infection or death among males. For instance, cardiovascular risk factors (heart disease, hypertension, and diabetes) and high-risk behaviors (social isolation, tobacco-smoking, alcohol use, and certain occupational exposures) are mostly associated with male gender [16]. Female sex hormones may also influence immune response regulation. Experimentally, it has been demonstrated that female mice were less prone to develop SARS-CoV infection than males. Further, increased mortality rate was increased among ovariectomized mice or female mice treated with estrogen receptor antagonist. It was concluded that estrogen receptor signaling may have a protective effects against SARS-CoV infection in females [17].

Age was a further risk factor for evolution of COVID-19, and the fifth decade may represent a critical age. Further, the results demonstrated that age can be considered as a death-associated risk factor. Most of Chinese data in this context agree that the infection was mostly observed in cases with advanced ages. Among 32,583 laboratory-confirmed COVID-19 cases from Wuhan (China), the median age of patients was 56.7 years, and elderly patients were at a higher risk of having severe or critical illness [13]. In a further Chinese study, 52 critically ill adult COVID-19 patients were explored. Their mean age was 59.7 years, and 61.5% of them died 28 days post-infection [14]. In a report from Korea, it has been found that age of COVID-19 patients showed M shape with two age peaks: 20s and 50s [18]. These findings might be expected because elderlies tend to have a higher prevalence of chronic diseases (for instance, cardiovascular diseases and diabetes) [19]. Further, reduced production of B and T cells in primary lymphoid organs and declined function of mature lymphocytes in secondary lymphoid tissues have been associated with aging [20]. These consequences will certainly increase the morbidity and mortality rates caused by viral and bacterial infections (including COVID-19) in elderlies.

Besides age and gender, ABO blood groups may also serve as susceptibility biomarkers for COVID-19. In this study, the overall distribution of the four phenotypes (A, B, AB, and O) showed a significant variation between patients (all cases, recovered, and deceased) and controls. In terms of individual phenotype, each group of patients was presented with specific profile. Among all cases of COVID-19, logistic regression analysis depicted an OR of 3.10 (95% CI 1.59–6.05) for group AB; therefore, such analysis suggested the susceptibility potential of this phenotype in the evolution COVID-19. Whereas, recovered cases were in favor of no significant association with ABO blood group phenotypes. On the contrary, deceased cases were markedly associated with group A (OR = 14.60; 95% CI 2.85–74.88). However, the three groups of patients shared a decreased frequency of group O, and the protective potential of such phenotype against evolution of COVID-19 was suggested. Recent studies have also depicted the significance of group O in lowering COVID-19 risk [4, 5, 10]. However, the three groups of investigators demonstrated the significance of groups A, B, or AB in increasing the risk of infection. Zhao and colleagues demonstrated further that group A was associated with a higher risk of death [5]. Together, these findings suggest that ABO antigens may interplay with pathogenesis of COVID-19; however, the mechanism(s) by which these molecules confer susceptibility or protection is subjected to speculations.

It has been speculated that infectious agents may influence human genome evolution through natural selection of specific alleles that may prone the population to the risk of infection. Further, these agents often use glycosylated cell-surface receptors for their successful attachment, and by such pathway, ABO determinants may affect host-pathogen interactions through utilization of glycosylation [7]. In SARS-CoV infection, it has been demonstrated that O-glycosylation plays a fundamental role in the virus pathogenesis [21]. Natural occurring anti-A and anti-B antibodies may also influence susceptibility to COVID-19 infection. In SARS-CoV infection, it has been hypothesized that these antibodies may decrease the rate of infection, and the degree of protection, may be influenced by the ABO antibody titer, secretor status, and incidence of group O in the population [9].

## Conclusions

The findings of this study suggest the significance of ABO blood group system in susceptibility to or protection against COVID-19. In this context, group AB may be a susceptibility biomarker for the infection, while group A may be associated with a higher risk of death. However, the present study was limited by the sample size especially in deceased cases. Thus, the obtained results motivated us to plan for a larger-scaled study based on a larger sample size of patients from Baghdad and other Iraqi cities.

## Data Availability

The datasets used and/or analyzed during the current study are available from the corresponding author on reasonable request.

## Abbreviations

CI: Confidence interval
COVID-19: Coronavirus disease 19
HWE: Hardy-Weinberg equilibrium
LSD: Least significant difference
OR: Odds ratio
*p*: Probability
*pc*: Bonferroni corrected *p*
SARS-CoV-2: Severe acute respiratory syndrome coronavirus 2
SD: Standard deviation
